# Improvement of Daptomycin Production in *Streptomyces roseosporus* through the Acquisition of Pleuromutilin Resistance

**DOI:** 10.1155/2013/479742

**Published:** 2013-09-10

**Authors:** Linli Li, Tingmei Ma, Qing Liu, Yuqi Huang, Changhua Hu, Guojian Liao

**Affiliations:** ^1^Institute of Modern Biopharmaceuticals, School of Pharmaceutical Sciences, Southwest University, Chongqing 400715, China; ^2^Chongqing Engineering Research Center for Pharmaceutical Process and Quality Control, Southwest University, Chongqing 400715, China

## Abstract

Daptomycin, a cyclic lipopeptide antibiotic produced by *Streptomyces roseosporus*, displays potent activity against a variety of gram-positive pathogens. There is a demand for generating high-producing strains for industrial production of this valuable antibiotic. Ribosome engineering is a powerful strategy to enhance the yield of secondary metabolites. In this study, the effect of a diterpenoid antibiotic pleuromutilin resistance mutation on daptomycin production was assessed. Spontaneous pleuromutilin-resistant derivatives of *S. roseosporus* were isolated. Sequencing of *rplC* locus (encoding the ribosomal protein L3) showed a point mutation at nt 455, resulting in the substitution of glycine with valine. G152V mutants showed increased production of daptomycin by approximately 30% in comparison with the wild-type strain. Its effect on daptomycin production was due to enhanced gene transcription of the daptomycin biosynthetic genes. In conclusion, pleuromutilin could be used as a novel ribosome engineering agent to improve the production of desired secondary metabolites.

## 1. Introduction

**Figure 1:**
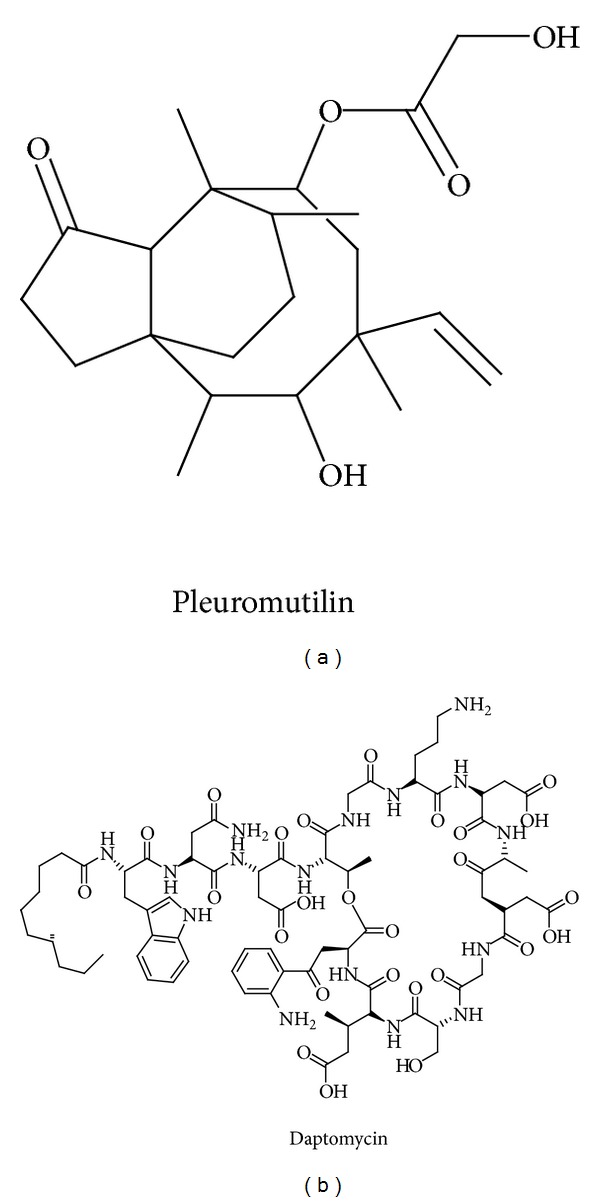
Chemical structures of pleuromutilin and daptomycin.

Daptomycin (Figure 1) is a cyclic lipopeptide produced by* Streptomyces roseosporus*. It shows excellent activity against Gram-positive pathogens, including methicillin-resistant *Staphylococcus aureus *(MRSA) or vancomycin-resistant *Enterococci* (VRE) [1]. Daptomycin has been approved for use in skin, skin-structure infections, and right-side endocarditis caused by* S. aureus* [2]. Daptomycin is a member of the A21978C factors consisting of 13 amino acids and a fatty acid which ranges from 10 to 13 carbon atoms [3]. Due to its pharmacological importance, considerable attention has been paid to the enhancement of the yield of daptomycin [4–7].

Ribosome engineering has been proved to be an efficient way for enhancing the production of secondary metabolites in a wide range of structural classes from a variety of actinomycetes strains [8]. Mutants could be easily obtained by simply screening resistant strains on drug-containing plates. Antibiotics targeting bacterial ribosome, such as streptomycin, gentamicin, and erythromycin, or targeting bacterial RNA polymerases, such as rifamycin, have been widely used to generate mutants with enhanced production of desired secondary metabolites [9–12]. Previous study in our lab demonstrated that K43N mutant in ribosomal protein S12 of *S. roseosporus* led to increased A21978C production by approximately 2.2-fold compared with the wild-type strain [13]. 

 Pleuromutilin is a diterpenoid antibiotic that acts by targeting large subunit of the bacterial ribosome and interacts with the peptidyl transferase center [14]. Resistance to pleuromutilin due to ribosome mutation has been demonstrated in a limited number of organisms [15–18]. However, the effect of pleuromutilin resistance mutation on antibiotic production in *Streptomyces* has not been reported. Therefore, we examined whether the acquisition of resistance to pleuromutilin enabled *S. roseosporus* to overproduce daptomycin.

## 2. Materials and Methods

### 2.1. Bacterial Strains and Growth Conditions

Bacterial strains used in this study are listed in Table 1. *Streptomyces roseosporus* ploxp, a producer of daptomycin, contained a reporter system which facilitated the selection of daptomycin overproducing strains [13]. *Micrococcus luteus* was used as the indicator strain for daptomycin bioassay.

Ploxp and its derivative strains were grown at 28°C in different media. Solid medium AS-1 and liquid medium TSB were prepared as described elsewhere [19]. F10A medium (CaCO_3_ 0.3%, distillers soluble 0.5%, soluble starch 2.5%, yeast extract 0.5%, glucose 0.5%, bactopeptone 0.5%) was used for daptomycin production. Decanoic acid (1% V/V in methyl oleate) was fed to the shake flask during fermentation to produce daptomycin.

### 2.2. DNA Manipulations

Molecular biology techniques were performed as described previously [20, 21]. Enzymes were purchased from Takara or TransGen and used according to the manufacturers' instructions. The FastPfu PCR system (TransGen) was used in PCR. Oligonucleotides (Table 2) were purchased from Invitrogen. DNA sequencing was carried out by the BGI technology.

### 2.3. Generation and Selection of Pleuromutilin-Resistant (Ple^r^) Strains

10^16^ spores of pSRE were spread out on AS-1 plate containing various concentrations of pleuromutilin. The resulting spontaneous Ple^r^ mutants were dotted on plates containing various amounts of kanamycin and 20 *μ*g mL^−1^ apramycin and then incubated at 28°C for three days. The resulting kanamycin-resistant mutants were used for further fermentation study. In the meantime, genomic DNAs were extracted from these mutants and used as templates for the amplification of *rplC* and 23S rRNA DNA fragment by PCR with primer pairs rplCF/rplCR and 23rRNAF/23rRNAR.

### 2.4. Fermentation of *S. roseosporus* and Daptomycin Bioassay

Spores of *S. roseosporus *and its derivatives were inoculated in TSB. The cultures were grown at 28°C on a rotary shaker (220 rpm) for 48 h and used as seed culture. One mL (2% V/V) of seed culture was inoculated into flasks containing 50 mL of F10A medium and then fermented at 28°C on a rotary shaker (220 rpm) for 6 days. The culture filtrates harvested by centrifugation were used for the determination of the cell dry weight and daptomycin bioassay as described [5]. To determine the cell dry weight, 10 mL cell cultures were collected by centrifugation. The cell pellet was washed with distilled water, collected by centrifugation, and dried at 60°C to constant weight.

## 3. Results

### 3.1. Isolation and Characterization of Spontaneous Ple^r^**** *S. roseosporus* Mutants

Spontaneous* S. roseosporus* mutants resistant to pleuromutilin were rarely isolated. From approximately 4 × 10^16^ spores, only 42 colonies were formed on AS-1 agar supplemented with 150 *μ*g mL^−1^ or 250 *μ*g mL^−1^ of pleuromutilin, which corresponds to approximately 3- to 5-fold amount of minimum inhibitory concentration (MIC). Spontaneous Ple^r^ mutants could be isolated at a frequency of approximately 10^−15^.

 Pleuromutilin binds to the ribosomal peptidyl transferase center. Spontaneous pleuromutilin resistance is often associated with mutations in ribosomal protein subunits or rRNA which prevent the antibiotic from binding to the ribosome. Common examples of these mutations include substitution of Asn148 of the L3 ribosomal protein subunit, which is encoded by the *rplC* gene or nucleotide positions 2032, 2055, and 2447 of the 23S rRNA [17, 18]. To investigate the basis of pleuromutilin resistance in *S. roseosporus*, we randomly selected 10 Ple^r^ strains and extracted their genomic DNA as the temple for PCR amplification. The *rplC* and 23rRNA gene loci were amplified and sequenced, respectively. No mutations in 23S rRNA were observed, whereas 8 Ple^r^ strains carried a single point mutation in the *rplC *gene which would lead to an amino acid change at codon 455, resulting in substitution of glycine with valine, and 2 Ple^r^ mutants harbored uncharacterized mutations outside of *rplC* and 23S rRNA (Table 3). As expected, all 10 strains exhibited little or no resistance to streptomycin or rifapicin (data not shown). 

### 3.2. Daptomycin Production by Ple^r^ Mutants

**Figure 2:**
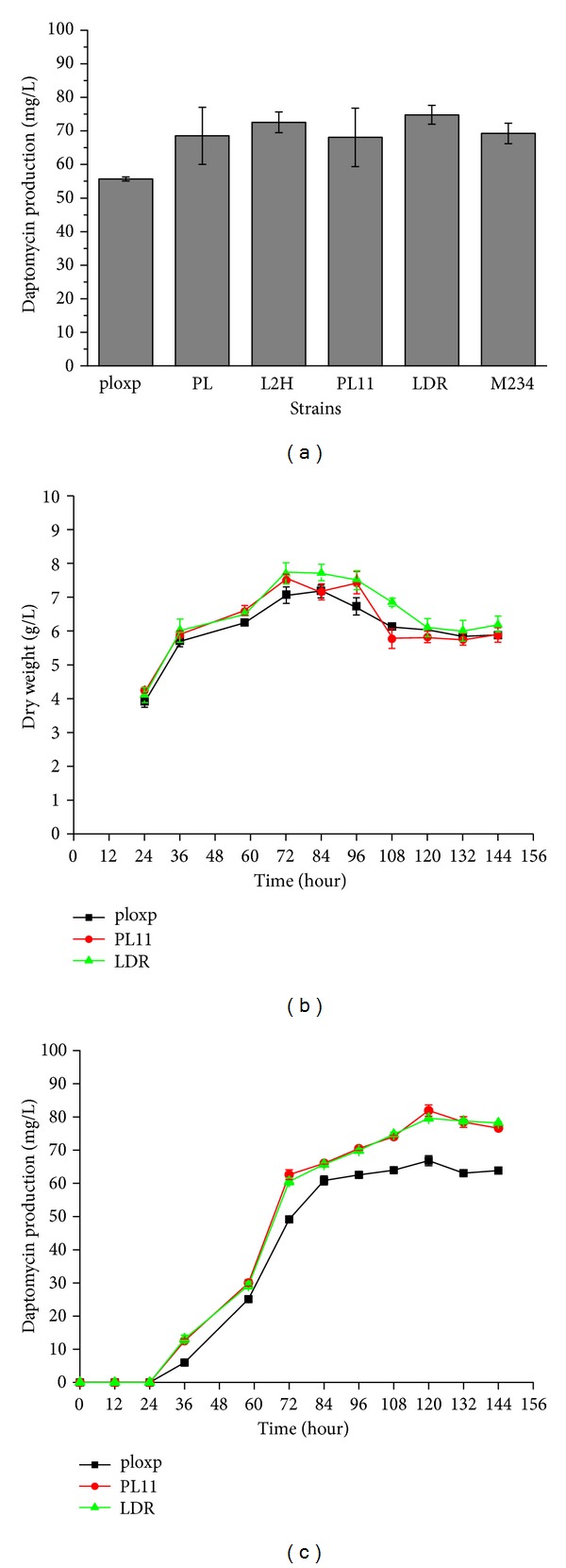
Daptomycin production and biomass of *S. roseosporus* and Ple^r^ mutants. (a) Daptomycin production in *S. roseosporus* and Ple^r^ mutants at 6 days; (b) cell dry weight; (c) daptomycin production in ploxp, PL11, and LDR. Data are presented as the averages of the results of three independent experiments. Error bars show standard deviations.

The effects of *rplC* mutation and the unknown mutation on daptomycin production in fermentation cultures were assessed. 3G152V mutants and 2 Ple^r^ mutants with unknown mutation sites were cultured in F10A liquid medium for 6 days and then the titer of daptomycin was measured. All 5 strains produced enhanced amounts of daptomycin compared to the wild-type strain (Figure 2(a)). These results suggest that different types of pleuromutilin resistance mutations positively affected the ability of *S. roseosporus* to produce daptomycin.

 To further clarify the effect of *rplC* mutation on fitness of the organism and daptomycin production. PL11 with G152V mutation, LDR with unknown mutation site, and wild-type strains were cultured in fermentation medium and were measured the daptomycin titer and cell mass during the entire time course. The two strains had comparable growth rates and final cell densities, indicating that higher cell density was not the mechanism for daptomcyin overproduction (Figure 2(b)). PL11 and LDR produced daptomycin in higher amount than the wild-type strain during the entire time course (Figure 2(c)).

To investigate the stability of PL11, the mutant was incubated in the absence of pleuromutilin. After 5 passages, 5 colonies were randomly selected and cultured in the presence of pleuromutilin for 5 days (about 150 generations). All of them still conferred pleuromutilin resistance and produced 1.3-fold of yields of daptomycin compared with the parent strain (data not shown). These results demonstrated that a PL11 mutant is genetically stable.

### 3.3. Effect of *rplC* Mutations on Gene Expression of Daptomycin Biosynthetic Genes

**Figure 3:**
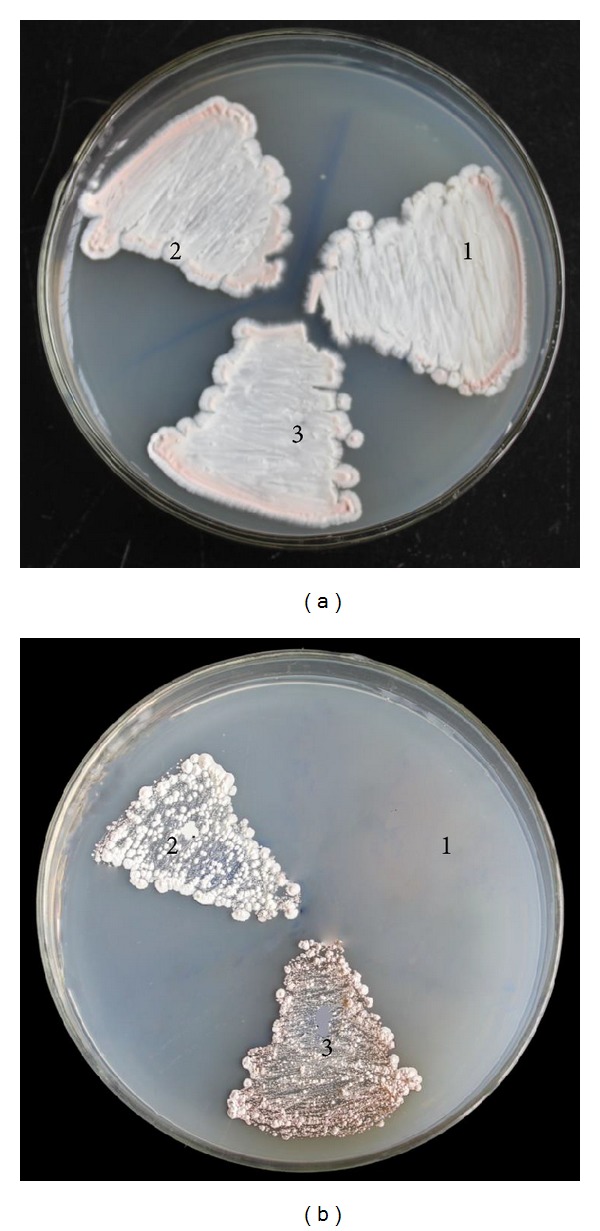
Kanamycin resistance level among *S. roseosporus *and its derivatives. (1) ploxp, a strain containing a reporter system in which *pdptE* was inserted in front of *neo*; (2) PL11, Ple^r^ were derivatives of ploxp with *rplC* mutation; (3) LDR, Ple^r^ derivatives of ploxp-harboring uncharacterized mutation site. (a) Without kanamycin; (b) 400 *μ*g mL^−1^ kanamycin.

To determine the effect of *rplC* mutation on transcription of daptomycin gene cluster, a transcription reporter system was used. ploxp and PL11 contained the chromosomal *pdptE::neo* transcriptional reporter fusion [13]. This reporter system was based on the assumption that the expression level of antibiotic biosynthetic genes positively correlated with the titer of daptomycin. The kanamycin resistance ability was measured and PL11 displayed higher kanamycin-resistant level than the wild-type strain, implying that the gene expression of *dptE* was enhanced (Figure 3). As the whole gene cluster ranging from *dptE* to *dptJ *and the contiguous genes from *dptE* to *dptH* may be transcribed on a giant polycistronic transcript [22], *rplC* mutations could result in increased gene transcription of the whole daptomycin gene cluster, which accounts for the observed effect of *rplC* mutation on daptomycin production.

## 4. Discussion

A mutation that confers to resistance to a drug targeting bacterial ribosomes, such as streptomycin, gentamicin, and paromomycin, is a powerful approach to enhancing the production of secondary metabolites in a wide range of structural classes [23]. In this study, we assess the beneficial effect of pleruomutilin on daptomycin production. The pleuromutilin classes of antibiotics are protein synthesis inhibitors that target the 50S subunit of bacterial ribosome. It was used for veterinary applications for more than 30 years and was recently approved for human use in 2007 [24]. Both the G152V mutant and mutants with uncharacterized mutation site increased the daptomycin significantly and produced daptomycin in higher amount than the wild-type strain during the entire time course, demonstrating that pleuromutilin could be used as a novel agent for ribosome engineering. 

Pleuromutilin was characterized by a low spontaneous mutation frequency of 10^−9^-10^−10^ against* S. aureus* and other organisms tested [17]. It was far more difficult to isolate spontaneous mutation in* S. roseosporus*, at a frequency of approximately 10^−15^. We tried a couple of times to isolate Ple^r^ mutants from *S. coelicolor* using as high as 10^17^ spores, but unfortunately no mutants were obtained, demonstrating that pleuromutilin had a very low spontaneous mutation frequency in *Streptomyces*. It must be pointed out that the extremely low mutation rate in *S. roseosporus* was unusual and may result from secondary mutation in the genome to compensate the lethal effect of *rplC* mutation. Further investigations such as whole genome sequencing are required to elucidate the mechanism underling the pleuromutilin resistance.

A number of lines of evidence showed that Ple^r^ *S. aureus* and other pathogens were predominantly linked to *rplC* mutations rather than to 23S rRNA mutation [18]. L3 protein consisted of about 210 amino acids and its mutation resistant to pleuromutilin centered to C-terminal. For example, G155R, D159Y, or S158L changes in the L3 protein were identified in *S. aureus* [17]. In this study, we identified a novel site G152V, which would enrich the mutation site pools and facilitate revealing the mechanism of action of pleuromutilin with ribosome. In this study, the overproducing phenotype in PL11 can be ascribed to a point mutation in the* rplC* and can be transferred to another high producer by genetic manipulation. Moreover, the resulting high-producing strains could be used as a starting strain to perform further ribosome engineering, as indicated where cumulative drug resistance mutations could dramatically enhance antibiotic production in *Streptomyces*.

## 5. Conclusion

 In this paper, we examined the effect of pleuromutilin resistance on antibiotic production in *Streptomyces*. The results showed that Ple^r^ mutants could increase the production of daptomycin by approximately 30%. Genetic analysis identified a novel G152V mutation site in L3 protein. Taken together, these results indicated that pleuromutilin can be used as a novel agent for ribosome engineering to enhance the production of secondary metabolites.

## Figures and Tables

**Table 1:** Bacterial strains used in this study.

Strains	Description	Reference
*Streptomyces roseosporus* ploxp	Wild-type strain containing a reporter plasmid in which *pdptE* was inserted in front of *neo *	[13]
*S. roseosporus* PL	*rplC* G152V, Ple^r^ derivative of ploxp	This study
*S. roseosporus* L2H	*rplC* G152V, Ple^r^ derivative of ploxp	This study
*S. roseosporus* PL11	*rplC* G152V, Ple^r^ derivative of ploxp	This study
*S. roseosporus* LDR	Ple^r^ derivative of ploxp with unknown mutation site	This study
*S. roseosporus* M234	Ple^r^ derivative of ploxp with unknown mutation site	This study
*Micrococcus luteus *	Daptomycin-sensitive indicator strain	[13]

**Table 2:** Primers used for PCR amplification of target genes.

Primer name	Description	Sequence
rplCF	Primes upstream of *rplC *	GGTGAACAAGCCCCTCAAG
rplCR	Primes downstream of* rplC *	GGTCAGGTTCTGGGTGGTG
23rRNAF	Primes upstream of 23S rRNA	AGAGACCAGCGAGAAGCGACT
23rRNAR	Primes downstream of 23S rRNA	GAACGTAGCCAACCAGCCAT

**Table 3:** Daptomycin phenotypes and genotypes of *rplC* mutants.

Strain	Daptomycin phenotype	Codon 455 sequence	Corresponding Amino acid	Amino acid change
ploxp	Wild type	GGT	Glycine	None
PL, L2H, and PL11	Daptomycin overproducer	GTT	Valine	G152V
LDR, M234*	Daptomycin overproducer	GGT	Glycine	None

*Mutation site uncharacterized.
